# Knowledge, attitudes, and practices regarding diabetic retinopathy among patients with diabetes in Dongola, Northern State, Sudan, 2022: a cross-sectional study

**DOI:** 10.11604/pamj.2024.48.77.43765

**Published:** 2024-06-28

**Authors:** Mohammed Abumhadi, Othman Amin, Sigoud Mohammed, Anas Mohamedelkhair, Esra Osman, Mohamed Issak, Wadah Mohammed, Omer Abdelmajid, Asjed Abdoun, Mohamed Abdelaziz

**Affiliations:** 1 Faculty of Medicine and Health Science, University of Dongola, Dongola, Northern State, Sudan,; 2 Faculty of Medicine Health Science, Elrazi University, Khartoum, Sudan,; 3 Faculty of Medicine Health Science, Omdurman Islamic University, Khartoum, Sudan,; 4 Department of Internal Medicine, Faculty of Medicine Health Science, University of Dongola, Dongola, Northern State, Sudan

**Keywords:** Diabetes mellitus, diabetic retinopathy, knowledge, attitude, practice, barrier, Dongola, Sudan

## Abstract

**Introduction:**

diabetic retinopathy (DR) is a potential complication of diabetes mellitus (DM). Both are a significant public health burden globally, affecting millions of people. The aim of this study was to assess the knowledge, attitude and practice toward DR among patients with diabetes in Dongola, Northern State, Sudan.

**Methods:**

this was a cross-sectional hospital-based study conducted among patients with diabetes attending Al-Jemaih Diabetic Center, from September 2022 to June 2023. The data were collected through face-to-face interview using a semi-structured questionnaire. Data was analyzed using Statistical Package for Social Science (SPSS), version 26. Statistical tests like Chi-Square, logistic regression tests were performed to explore association and its strength, considering statistical significance at p≤0.05.

**Results:**

among the 241 patients who participated in the study, about two-third, 63.9% (n=154) being females. About half, 55.2% (n=133) had good DR knowledge, which was significantly associated with urban residence (aOR: 2.58, 95% CI: 1.45-4.62; p=0.001). Majority, 79.3% (n=191) reported favorable DR attitude. About two-third, 63.9% (n=154) reported good DR practice. However, only 27.8% (n=67) routinely go for eye check-up. Knowledge was significantly associated with practice (aOR: 2.18; 95% CI: 1.13-4.20; p=<0.019). The most common barrier reported hindering regular eye check-up was misconception that the eye check-up is not necessary, 39.4% (n=65).

**Conclusion:**

despite the good knowledge, favorable attitude and good practices, the regular eye check-up practice was significantly low. Urban residence was significantly associated with knowledge. Similarly, knowledge was found to be significantly associated with practice level. The most common barrier to regular eye check-up was the misconception that it is not important.

## Introduction

Diabetes Mellitus (DM) is a chronic metabolic disorder characterized by elevated blood glucose levels due to the body's inability to produce insulin, effectively utilize it, or both [1]. It is a significant public health burden globally, with an estimated 537 million adults (20-79 years) living with diabetes in 2021 [2]. This number is steadily increasing, projected to reach 643 million by 2030 and 783 million by 2045 [2]. The prevalence is more common in low and middle-income countries, where access to healthcare services and awareness about the condition may be limited [2]. Diabetes mellitus has potential consequences on various organs within the body, with diabetic retinopathy (DR) being one of its many potential microvascular complications [3]. In DR, small blood vessels in the retina are damaged by high blood glucose, leading to leakage, swelling, formation of abnormal new blood vessels, and if left untreated, vision loss and irreversible blindness [4]. The prevalence of DR is significantly high, with a global prevalence of 22.27% [5]. The prevalence of DR is highest in Africa (35.90%) [5,6]. In Sudan, older studies from 1991 and 1995 reported a DR prevalence of 17.2% and 43%, [7,8]. A recent study from Makka eye complex in Khartoum reported a significantly higher prevalence of DR at 82.6% [9]. Several risk factors for developing DR among patients with DM include prolonged duration of diabetes, poor glycemic control, hypertension, dyslipidemia, and genetic factors [10]. Conversely, several strategies for preventing it have been proposed, such as maintaining normal blood glucose, blood pressure, and cholesterol levels; exercising; and regular eye check-ups [11]. Regarding the knowledge, attitudes, and practices (KAP) of patients with diabetes towards DR, several studies reported variable rates. For instance, a study from Eastern India reported average good knowledge (56.3%), a positive attitude (68.0%), and poor DR practices (66.6%) [12] Another study from Northwest Ethiopia revealed an average level of good knowledge (47.4%) and poor practices (39.6%) [13]. Locally, a study from Khartoum reported poor eye check-up practices (35.5%) [14]. While DM is more prevalent in Dongola, no previous study has assessed the KAP of patients with diabetes towards DR there. This is crucial for developing effective strategies for prevention, early detection, and management of the condition. This study aimed to assess the KAP towards DR among patients with diabetes in Dongola and determine the common barriers hindering good practice.

## Methods

**Study design and setting:** the design used in the current study was a cross-sectional hospital-based study design, conducted between September 2022 and June 2023. The study setting was Al-Jemaih Diabetic Center, which is a large tertiary hospital that serves the patients with diabetes in the Northern State, Sudan. The study strictly adhered to the cross-sectional reporting guidelines of Strengthening of Reporting of Observational Studies in Epidemiology (STROBE) [15].

**Study population:** the study targeted patients with diabetes attending Al-Jemaih Diabetic Center for follow-up. Any adult (>18 years) diabetic patient, attending at Al-Jemaih Diabetic Center who provided consent for interview was included in the study. Patients with poor communication problems, severely ill, pregnant ladies, patients with mental illness, and those who did not give consent or complete the survey were excluded. A sample size was calculated using Rao soft sample size calculator and considering a confidence interval of 95%, a hypothesized prevalence of DM among the population of 16%, and margin of error of 5% as 207 [16]. It was increased up to 241 to compensate for low response rates, or incomplete responses. The sampling method was convenience sampling procedure.

**Study variables:** the independent study variables were the socio-demographic information which included sex, age, monthly income, educational level and residence. The dependent (outcome) variables were the knowledge, attitude, and practice of the participants towards DR. The both variables were summarized and presented inform of frequencies and percentages. Comparative analysis was conducted between the independent and outcome variables, while considering the possible confounders.

**Data collection:** a semi-structured questionnaire developed, modified and adopted from several previous studies was used to collect data [12-14]. It was translated in to Arabic for avoidance of bias in simultaneous translation variations among the investigators. The tool consisted of 5 sections: i) sociodemographic data of the participants, comprised of 5 domains; ii) knowledge dimension, consisting of 10 questions; iii) attitudes dimension, consisting of 10 statements; iv) practices dimension, consisting of 6 questions; and (5) other relevant information section, consisting of 5 questions. The questionnaire was piloted on 15 patients using the questionnaire to evaluate the overall suitability, understandability and clarity of the instrument for the participants. For content validation, two experts in the researched topic (a consultant in internal medicine, and a clinical biochemist) reviewed and approved the questionnaire. To determine the reliability of the questionnaire, Cronbach´s alpha coefficient was calculated, and was found to be 0.72, which is considered reliable [17]. The primary research data were collected via face-to-face interviews for over 6 months, in two separate times; from September to December 2022, and from March to May 2023. Prior to the interviews, participants were asked to answer each question honestly and based on the truth. A unique identification number was assigned for each data and to keep confidentiality, no personal identification information was collected.

**Statistical analysis:** the data were analyzed using the Statistical Package for Social Science (SPSS), version 26. Descriptive and comparative analyses were performed, and are presented in the form of tables and figures. For scoring purposes, a one-point score was assigned for each correct option, and a total score of 50% or more was considered good. The Chi-square test was conducted to assess the association between variables. Similarly, univariable and multivariable Logistic Regression tests were used to assess the magnitude of the associated variables, with adjusted odd ratio to control for confounding. Missing and incomplete responses were removed from the final analysis. All statistical tests were judged to be significant at a p-value ≤0.05.

**Ethical consideration:** the study was carried out in accordance with the Declaration of Helsinki. Ethical approval was obtained from the Research Ethical Committee of the Northern State Ministry of Health (Approval Number: 7/2022) and the research office of Al-Jemaih Diabetic Center. Informed written consent was obtained from all respondents prior to interviewing them.

## Results

**General characteristics of the study population:** a total of 241 fulfilling the inclusion criteria were interviewed for the study. About two-third (154 (63.9%) were females. Most of them aged 50-64 years 117 (48.5%), resided from rural residencies 146 (60.3%), had a moderate monthly income level of 100.00-500.00$ 174 (72.2%), and were educated up to primary level 86 (35.7%). The socio-demographic data are illustrated in Table 1.

**Table 1:** socio-demographics of the study participant, 2023

Characteristics	Frequency	Percent
**Sex**		
Male	87	36.1
Female	154	63.9
**Age (years)**		
< 50 years	60	27.4
50-64 years	117	48.5
>64 years	58	24.1
**Monthly income (in USD)**		
<100.00$	54	22.4
100.00 - 500.00$	174	72.2
>500.00$	13	5.4
**Educational level**		
Uneducated	58	24.1
Primary school	86	35.7
Secondary school	57	23.7
University	40	16.6
**Residence**		
Rural	146	60.3
Urban	96	39.7

**Knowledge of the study population about diabetic retinopathy:** regarding the knowledge, the first section addressed the general knowledge of the patients regarding DM. In the section, majority identified the correct test for diagnosing DM (69.3%), and the one that reflects control of blood sugar (76.8%). Similarly, majority acknowledged that DM is a treatable disease (85.1%), and requires life-long control (88.0%). However, only few correctly identified all the factors that help keep DM under control (22%). In the second section, the questions assessed the knowledge of the participants about DR. Most of them knew that DM affects the eye (74.7%), and that it causes retinopathy and cataract (53.1%). However, only few knew that once DR is formed, no normal vision restoring is possible (30.3%), that all DM should have periodic and regular eye check-up for DR (37.3%), and if not treated, it causes blindness (37.8%). Overall, the participants revealed average (55.2%) knowledge level about DR. This data is shown in Table 2.

**Table 2:** knowledge of the study participant towards diabetic retinopathy, 2023

Question	Frequency	Percent (%)
**What are the tests used in diagnosis of diabetes mellitus?**		
Blood test	59	24.5
Urine test	5	2.1
Both	167	69.3
Others	10	4.1
**What are the factors that help keeping diabetes mellitus under control?**		
Medication only	51	21.2
Diet only	117	48.5
Exercise only	14	5.8
Weight reduction only	6	2.5
Regular follow-up only	0	0
All above	53	22.0
**Diabetes mellitus is?**		
Curable	16	6.6
Treatable	205	85.1
Malignant	5	2.1
Self-limiting	3	1.2
None of the above	12	5.0
**Control of diabetes mellitus should be?**		
Until sugar level gets under control	13	5.4
Life-long	212	88.0
Any other	16	6.6
**Which test reflects control of blood sugar for a long period?**		
HbA1c test	185	76.8
Other	56	23.2
**Does diabetes mellitus affect eyes?**		
Yes	180	74.7
No	61	25.3
**What problems can patients with DM have in the eye?**		
Retinopathy & cataract	129	53.1
Infection	1	0.4
Others	51	21.2
No effect	61	25.3
**Can a person with DR have normal vision?**		
Yes	54	9.1
No	174	30.3
I don’t know	13	35.3
No effect	61	25.3
**Can DR cause blindness?**		
Yes	91	37.8
No	2	0.8
I don’t know	87	36.1
No effect	61	25.3
**Should patients with DM have regular eye check-up for DR?**		
Yes	90	37.3
No	5	2.1
I don’t know	85	35.3
No effect	61	25.3
**Overall Knowledge Score**		
Poor	108	44.8
Good	133	55.2

DR: diabetic retinopathy; DM: diabetes mellitus

**Attitude of the study population toward diabetic retinopathy:** in the attitude section, majority agreed to the importance of going regular check-up even if the blood glucose is under control (91.3%), and the possibility of keeping blood sugar under control (86.3%). Similarly, most of them agreed to going regular eye check-up even if the blood glucose is under control (71.4%) or there is no indication of eye problem (68.5%). In addition, majority disagreed skipping medications (64.7%), taking medications without prescription (85.9%), or replacing it with herbal medicines (68.5%). Less than half disagreed eating sweets (40.7%) and that normal blood glucose can be maintained by daily activities without exercise (38.2%). In general, most of the participants reported favorable attitude (79.3%) towards DR. Attitude assessment questions are illustrated in Table 3.

**Table 3:** attitude of the study participant towards diabetic retinopathy, 2023

Statement	Frequency	Percent (%)
**Options**	**Agree**	**Disagree**
It is ok to eat sweats from time to time	143 (59.3%)	98 (40.7%)
It is acceptable to skip some medications	85 (35.3%)	(64.7%)
Going to regular check-up even if a blood glucose is under good control	220 (91.3%)	21 (8.7%)
Daily activities without exercises are enough to maintain normal blood glucose	149 (61.8%)	92 (38.2%)
It is possible to keep blood sugar under control, thus preventing some of the complications that related to DM	208 (86.3%)	33 (13.7%)
Regular use of herbal medicine can replace ordinary hypoglycemic agents	74 (31.5%)	165 (68.5%)
It’s acceptable to take medications without physician prescription	34 (14.1%)	207 (85.9%)
Going to regular eye check-up even if a blood glucose is under control	172 (71.4%)	69 (28.6%)
Going to regular eye check-up even if there is no indicator for any eye problem	165 (68.5%)	76 (31.5%)
When eye vision becomes poor, its purposeless to take medication and continue regular checkup	51 (21.2%)	190 (78.8%)
**Overall attitude score**		
Negative	50 (20.7%)	
Positive	191 (79.3%)	

DM: diabetes mellitus

**Practice of the study population regarding diabetic retinopathy:** regarding the practice towards DR, majority reported taking medications as prescribed by their physicians (92.5%), not altering dose without physician consultation (74.3%), and on regular follow-up (83.0%). About half reported either working in high physical effort jobs (33.2%) or exercising sufficiently (19.1%). However, only about half (56.8%) reported going for regular eye check-up, and among them only few checked for the recommended frequency of once in three months (15.4%), or as advised per their physicians (12.4%). Generally, the overall practice level was good (63.9%). Table 4 demonstrates these aspects.

**Table 4:** practice of the study participant towards diabetic retinopathy, 2023

Question	Frequency	Percent (%)
**Do you take medications for Diabetes mellitus as advised by the physician?**		
Yes	223	92.5
No	18	7.5
**Does your job demand high physical effort or exercise sufficiently?**		
High physical effort job*	80	33.2
Sufficient exercise**	46	19.1
None	115	47.7
**Do you alter medication dose depend on your meal habits without physician consultation?**		
Yes	62	25.7
No	179	74.3
**Do you visit your physician for regular follow-up?**		
Yes	200	83.0
No	41	17.0
**Do you go for regular eye check-up?**		
Yes	137	56.8
No	104	43.2
**How often do you go for regular eye check-up?**		
Once every 3 months or as advised by the doctors	37	27.8
Once every 6 months	26	10.8
Once every year	44	18.3
None	104	43.2
**Overall practice score**		
Poor	87	36.1
Good	154	63.9

**Factors associated with poor knowledge, attitude and practice:** regarding the risk factors, (illustrated in Table 5, Table 6) urban residents were found to be two times more knowledgeable about DR than rural residents (aOR: 2.58, 95% CI: 1.45 - 4.62; p = 0.001). Knowledge was also significantly associated with practice level; participants with good knowledge about DR reported almost two times more good practice than those with poor knowledge (aOR: 2.18, 95% CI: 1.13-4.20; p=<0.019). Physicians were the most common source of information (51.0%). The self-reported prevalence of DR and other eye diseases were 14.9% and 36.5% respectively. Private clinics were the most common centre reported for eye check-up (41.8%). The most common barriers hindering the regular follow-up and eye check-up was misconception that it´s not important (38.8%) (Table 7) only few checked for the recommended frequency of once in three months (15.4%), or as advised per their physicians (12.4%).

**Table 5:** correlation of basic characteristics of the studied participants with diabetic retinopathy, knowledge, attitude, and practice, using Chi-Square test, 2023

Variable	Knowledge	Attitude	Practice
**Correlation**	**P-value**	**P-value**	**P-value**
**Sex**			
Male	0.059	0.728	0.910
Female			
**Age**			
<50 years	0.405	0.769	0.753
50-64 years			
>64 years			
**Educational level**			
Uneducated	0.227	0.085	0.478
Primary School			
Secondary School			
**Monthly income (USD)**			
<100.00$	0.225	0.140	0.984
100.00-500.00$			
>500.00$			
**Residence**			
Rural	<0.001*	0.228	0.146
Urban			
**Knowledge about DR**			
Poor	NA	0.037*	0.031*
Good			
**Attitude towards DR**			
Negative	NA	NA	0.009*
Positive			

*: Statistically significant**;** NA**:** not applicable**;** DR: diabetic retinopathy

**Table 6:** risk assessment of factors affecting the knowledge and attitude of the study participants towards diabetic retinopathy, using univariable and multivariable logistic regression test, 2023

Variable		cOR (95% CI)	P-value	aOR (95% CI)	P-value
**Knowledge**	**Residence**				
	Rural	Reference			
	Urban	2.68 (1.56-4.62)	<0.001*	2.58 (1.45-4.62)	0.001*
**Attitude**	**Knowledge about DR**				
	Poor	Reference			
	Good	1.96 (1.04-3.68)	0.037*	1.82 (0.94-3.54)	0.077
**Practice**	**Knowledge about DR**				
	Poor	Reference			
	Good	1.79 (1.05-3.05)	0.031*	1.61 (0.91-2.84)	0.098
	**Attitude towards DR**				
	Negative	Reference			
	Positive	2.39 (1.23-4.35)	0.009*	2.18 (1.13-4.20)	0.019*

*: Statistically significant**;** cOR**:** crude odd ration**;** aOR**:** adjusted odd ratio; DR: Diabetic retinopathy

**Table 7:** other relevant information from the study participant, 2023

Variable	Frequency	Percent (%)
Source of knowledge about DR*		
Physician	75	51.0
Television	21	14.3
Books	7	9.5
Neighbors	30	20.4
Others	14	4.8
**Diagnosed with DR?**		
Yes	36	14.9
No	200	83.0
Not sure	5	2.1
**Diagnosed with another eye disease?**		
Yes	88	36.5
No	149	61.8
Not sure	4	1.7
**Center for regular eye checkup?** *		
Public hospital	26	38.8
Private hospital	28	41.8
Specialized eye center	10	14.9
Other	3	4.5
**Barriers towards regular follow-up and eye check-up?** **		
Misconception that it is not important	65	39.4
Lack of trust in the physician	11	6.7
Far distance of the healthcare center	19	11.5
Financial problem	15	9.1
Busy timetable	21	12.7
Others	34	20.6

*: Multiple response analysis; **: multiple response analysis (Only those who reported non-compliance to regular follow-up and eye check-ups were included) DR: diabetic retinopathy

## Discussion

Diabetes mellitus and DR are growing burden that overwhelm both developing and developed countries, and unfortunately their incidences are on rise [2]. The current study aimed to explore the KAP of diabetic patients concerning DR at Al-Jemaih Diabetic Centre. The reported KAP levels were; average good knowledge (55.2%), positive attitude (79.3%), and good practice (63.9%). There was poor compliance to regular eye check-ups (27.8%), with the most common barrier reported being misconception that it is not important (39.4%). In the current study, 55.2% of participants had good knowledge about DR. This finding is consistent with similar studies from Northwest Ethiopia, and Saudi Arabia, which reported knowledge levels of 47.7%, and 52.9%, respectively [13,18], indicating a wider commonality in understanding DR among diabetic patients in these areas. The slightly higher percentage of knowledge in our study than in Ethiopia might reflect on differences in health education efforts or access to information. In contrast, two studies from Kenya and Yemen reported a significantly lower knowledge level of 33% and 32.2%, respectively [19,20]. The difference could be attributed to geographical, socio-cultural differences, and variations in the assessment tools used. Attitudes towards DR were notably positive in our study, with 79.3% of participants demonstrating a good attitude. This could be considered as a promising finding as positive attitudes are crucial for proactive health behaviors [21]. Similar positive attitude rates (80.8% and 83.1%) were reported in the studies from Saudi Arabia and Egypt, respectively [18,22]. However, this contrasts sharply with studies from Mozambique and Pakistan, which reported poor attitude levels of 48.5%, and 27.5% (0.55± 0.15 out of a score of 2), respectively [22,23]. The disparity in attitude levels could be due to variations in the effectiveness of public health campaigns, healthcare access, and the overall health literacy of the populations studied.

Regarding practice, 64% of participants reported good overall practice scores. Similar good practice rates (66.6%) were reported in the study from Eastern India [12]. In contrast, the study from Northwest Ethiopia reported a lower good practice rate of 39.6% [13]. However, with this good practice rate in the current study, only 27.8% adhered to regular eye check-up practices. Similar low adherence rate (31%) was reported in a local study from Khartoum, indicating a local burden [14]. The lower adherence to regular eye check-ups is a significant concern because regular monitoring is essential for early detection and management of DR. The gap between general good practice and specific eye check-up practices suggests that while patients might adhere to general diabetes management routines, they may not fully recognize the importance of regular eye check-ups. This highlights the importance of targeted educational campaigns that emphasize the critical role of regular eye examinations in preventing vision loss. A study conducted in Egypt reported that diabetic patients' practice and attitude significantly improved after attending the health education sessions [24]. Urban residence was significantly associated with higher knowledge levels (aOR: 2.58, 95% CI: 1.45 - 4.62; p = 0.001) in the current study, a finding consistent with the study from Northwest Ethiopia (aOR: 2.65, 95% CI: 1.16 - 6.07; p = <0.05) [13]. Urban residents often have better access to healthcare facilities, educational resources, and media that disseminate health information, which likely contributes to their higher knowledge levels. This urban-rural disparity underscores the need for focused health education and outreach programs in rural areas to bridge the knowledge gap. In addition, attitude was found to be significantly correlated with the practice level. Participants with a positive attitude towards DR were more than two times more likely to have better DR practices (aOR=2.18; 95% CI: 1.13-4.20; p=<0.019). These findings are consistent with those of the study from Eastern India and underscore the interconnectedness of KAP elements [12]. Promoting knowledge through education can positively influence and improve DR practices.

Regarding the barriers and low compliance to regular follow-up and eye check-up, the most common one identified in the current study was the misconception that regular check-ups are unnecessary (39.4%). This misconception was also reported in several studies from Khartoum, Kenya, and Pakistan, and highlights a critical area for intervention [14,19,23]. Misconceptions about the necessity of regular check-ups can lead to delayed diagnosis and treatment of DR, resulting in preventable vision loss. Addressing these misconceptions through targeted educational interventions is essential. Healthcare providers should emphasize the importance of regular eye examinations, regardless of the current state of blood glucose control, to prevent or manage DR effectively. Physicians were the main source of information for the patients in the current study (51%), which is consistent with studies from other regions, such as Khartoum, and Northwest Ethiopia [14,13]. These findings emphasize the crucial role that healthcare providers, play in educating patients about diabetes management and prevention of complications. The prevalence of DR in the current study was 14.9%, which aligns with the reported prevalence in the study of Khartoum, which was 13% [14]. This suggests that the burden of DR among patients with diabetes is relatively consistent across the country, highlighting the importance of regular eye screenings for individuals with diabetes to detect and manage this complication early. Additionally, the main center for regular eye check-ups reported in the current study being private clinics (41.8%) is similar to the findings from the study of Khartoum, where 51% of patients also sought eye care at private clinics [14].

Strengths of the current study include the use of a content-validated and statistically reliable questionnaire, and a well-representative sample size. The content validity and statistical reliability of the questionnaire ensure that the findings are robust and reflective of the study population's actual KAP regarding DR. However, there are several limitations. The cross-sectional design limits the ability to establish causal relationships between knowledge, attitudes, and practices. Additionally, the reliance on self-reported data introduces the potential for response bias, although this was mitigated by conducting face-to-face interviews. In addition, there is temporal limitation as well, which may not allow continuous generalization of the findings, emphasizing the need for continuous monitoring and research to keep up with changes in KAP among patients with diabetes. Based on the findings in the current study, the researchers recommend addressing the reported barriers through providing comprehensive health education, and organizing routine, active awareness-raising campaigns and community-based strategic interventions regarding DR. Researchers also recommend further studies and interventions to promote eye check-up practices among the patients with diabetes.

## Conclusion

The current study assessed the KAP among the patients with diabetes at Al-Jemaih Diabetic Center towards DR. Most participants demonstrated good knowledge, favourable attitude and good practices. However, the regular eye check-up practice was significantly low. Urban residence was significantly associated with good knowledge about DR. Similarly, knowledge was found to be significantly associated with practice level. The most common barrier to regular eye follow-up was the misconception that it is not important.

### What is known about this topic

- *Diabetic retinopathy is a serious complication of diabetes mellitus with a global prevalence of 22.27%;*
- *Knowledge, attitudes, and practice rates towards diabetic retinopathy among the patients with diabetes revealed variations, most probably due to geo-socio-cultural differences;*
- *High proportion of patients with diabetes do not comply with DR screening protocol, mainly because of poor knowledge*.

### What this study adds

- *The knowledge, attitudes, and practices towards diabetic retinopathy among the patients with diabetes from Al-Jemaih Diabetic Center were 55.2%, 79.3%, and 64%, respectively;*
- *Despite the average knowledge (55.2%), favourable attitudes (79.3%) and good practices (64.0%, only 27.8% reported going for regular eye check;*
- *The most common barrier to regular eye check-up was the misconception that it is not important (39.4%)*.
